# Gene expression-based enrichment of live cells from adipose tissue produces subpopulations with improved osteogenic potential

**DOI:** 10.1186/scrt502

**Published:** 2014-10-06

**Authors:** Hetal D Marble, Bryan A Sutermaster, Manisha Kanthilal, Vera C Fonseca, Eric M Darling

**Affiliations:** Department of Molecular Pharmacology, Physiology, and Biotechnology, Brown University, Providence, RI 02912 USA; Center for Biomedical Engineering, Brown University, Providence, RI 02912 USA; Department of Orthopaedics and School of Engineering, Brown University, Providence, RI 02912 USA

## Abstract

**Introduction:**

Mesenchymal stem cells have been increasingly used for cell-based therapies. Adipose-derived stem/stromal cells (ASCs) from the stromal vascular fraction (SVF) of fat tissue are a particularly attractive option for cell based therapy given their accessibility and relative abundance. However, their application in both clinical and basic science investigations is complicated by the isolation of differentiable cells within the SVF. Current enrichment strategies, such as monolayer passaging and surface marker-based sorting, can be time-consuming or overly stringent. Ideally, a population of cells with great regenerative capacity could be isolated with high yields so that extensive *in vitro* manipulation is not necessary. The objective of this study was to determine whether SVF cells sorted based on expression of alkaline phosphatase liver/bone/kidney (*ALPL*) resulted in populations with increased osteogenic differentiation potential.

**Methods:**

SVF samples were obtained from four, human donors and processed to isolate initial, heterogeneous cell populations. These SVF cells underwent a four day osteogenic priming period, after which they were treated with a fluorescent, oligodeoxynucleotide molecular beacon probe specific for *ALPL* mRNA. Cells were separated into positive and negative groups using fluorescence-activated cell sorting (FACS) then differentiated down the osteogenic lineage. Differentiation was assessed by measuring calcified matrix production in each sample.

**Results:**

Cells positive for *ALPL* expression (*ALPL*+) represented approximately 34% of the gated population, while cells negative for *ALPL* expression (*ALPL-*) represented approximately 18%. *ALPL*+ cells produced 3.7-fold and 2.1-fold more calcified matrix than *ALPL*- and unsorted SVF cells, respectively, indicating a significant improvement in osteogenic differentiation. Further, *ALPL*+ cells showed increases in metabolite production for both adipogenesis and chondrogenesis, suggesting that the enrichment process yields an enhanced multipotent phenotype. Osteogenic differentiation response and cell yields for *ALPL+* cells were markedly improved over surface marker-sorted samples.

**Conclusion:**

This study demonstrates a novel method to enrich heterogeneous SVF cells for increased osteogenic potential. The procedure requires less time and results in higher yields of therapeutically useful cells than other existing approaches. Gene expression-based sorting of MSCs is a potentially paradigm-shifting approach that could benefit applications spanning from basic science to clinical therapy.

**Electronic supplementary material:**

The online version of this article (doi:10.1186/scrt502) contains supplementary material, which is available to authorized users.

**Box 1:**
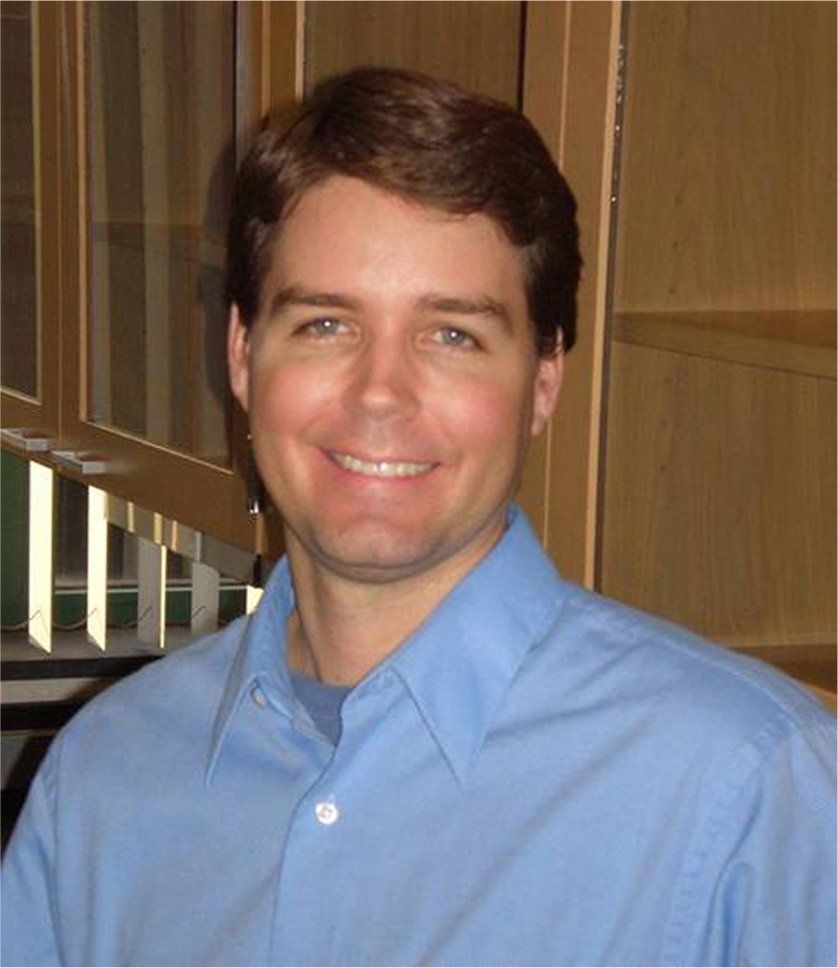
About Eric Darling **EMD** is the Manning Assistant Professor of Medical Science, Orthopaedics, and Engineering in the Department of Molecular Pharmacology, Physiology, & Biotechnology at Brown University. He also currently serves as the Graduate Program Director for the Center for Biomedical Engineering. He received a BS in engineering from Harvey Mudd College, a PhD in bioengineering from Rice University, and postdoctoral training in orthopedic research at Duke University. His research area focuses on understanding the relationship between the mechanical and biological characteristics of cells and tissues, with emphasis on the musculoskeletal system. He is specifically interested in understanding heterogeneity in adult stem cell populations and developing approaches to identify tissue-specific cells for regenerative medicine and disease diagnostics. Recent work in his group has focused on two, novel cellular characteristics: single-cell mechanical biomarkers and live-cell gene expressions.

## Introduction

Cell enrichment or purification is often a necessary first step for clinical, cell-based therapies as well as basic science investigations into homogeneous subpopulations. Adult mesenchymal stem cells (MSCs) are one type of cell for which this is of great importance. As our understanding of MSCs improves, their use in regenerative medicine becomes ever more promising. This has been especially true for musculoskeletal tissues, with researchers proposing many possibilities using MSCs for the treatment of orthopedic ailments [1–7]. Autologous stem cell transplantation therapies have been proposed for their potential therapeutic versatility and low immunogenicity [8, 9]. However, many of these proposed therapies rely on prior *in vitro* expansion of the cell populations, which is a slow process and can negatively affect cell phenotype [10]. Single-surgery therapies, where autologous MSCs are isolated and reintroduced into the site of injury in short succession, have the potential to save on both costs and rehabilitation time. Human adipose-derived stem/stromal cells (ASCs), isolated from the stromal vascular fraction (SVF) of lipoaspirate, may be particularly well suited for such single-surgery strategies due to their accessibility and relative abundance in fat tissue, as well as their ability to differentiate down the osteogenic, adipogenic, and chondrogenic lineages [11].

While prolonged culture and passaging is an effective method for isolating ASCs from SVF populations, this process can take weeks to complete. A more rapid approach for the isolation of regeneratively advantageous cells from other cell types contained within the SVF would be enormously beneficial. Traditional cell enrichment strategies have found limited success purifying MSCs due to the lack of a universal surface marker profile [12]. This approach is further complicated by the temporal variability of surface antigens, which can change over time with passaging [13]. Additionally, reported surface marker profiles often result in very low cell yields, necessitating post-sort expansion to obtain sufficient cell numbers for practical applications [14]. Discarded SVF cells can include mesenchymal and endothelial progenitors that may have the potential to differentiate down a subset of lineages [15, 16]. Rigorous surface marker definitions that have been proposed in the literature or by organizations such as the International Federation for Adipose Therapeutics and the International Society for Cellular Therapy may be unnecessarily restrictive for therapeutic applications since they exclude a large fraction of cells with regenerative potential [17].

Alternative enrichment strategies are needed to sort large numbers of therapeutically beneficial cells from the SVF. We thus propose a cell sorting scheme based on expression of mRNA, using molecular beacons as fluorescent reporters. A molecular beacon is an oligodeoxynucleotide, hairpin-shaped, hybridization probe with a fluorophore on the 5′ end and a quencher on the 3′ end [18]. The probe fluorescence is quenched in the absence of target oligonucleotide but is unquenched when the loop region binds to its target mRNA. Other groups have used molecular beacons in conjunction with fluorescence-activated cell sorting (FACS) to sort embryonic stem cells based on *OCT4* and *SOX2* expression for stemness and pluripotent stem cells based on *MHC* and *TNNT* gene families for cardiomyogenesis [19–21]. Given previous successes using molecular beacons with pluripotent stem cells, we apply them here to obtain therapeutically useful cells from a heterogeneous mesenchymal cell population, the SVF.

The objective of this study was to sort cells derived from the SVF of adipose tissue based on expression of alkaline phosphatase liver/bone/kidney (*ALPL*) to obtain subpopulations of cells capable of enhanced osteogenesis. To do this, we employed a custom-designed molecular beacon for *ALPL* in combination with FACS [22]. This approach produced high-yield isolations of cellular subpopulations capable of significantly enhanced osteogenesis compared with both unsorted SVF cells and surface-marker sorted ASCs, suggesting increased therapeutic potential for bone regeneration therapies.

## Materials and Methods

### Cell isolation, culture, and multipotency assessment

#### Media compositions

Cells were cultured in defined media that served to either maintain stemness, act as a control condition, or induce a differentiation response. Stromal medium, which acted as a control condition, contained Dulbecco’s modified Eagle’s medium (DMEM) with Ham’s F12 salt solution in a 1:1 ratio, 10% fetal bovine serum (FBS) (Zen-Bio, Research Triangle Park, NC, USA), and 1% antibiotic/antimycotic (Fisher Scientific, Pittsburgh, PA, USA). Expansion medium contained stromal medium, with the addition of 5 ng/ml epidermal growth factor, 1 ng/ml fibroblast growth factor, and 0.25 ng/ml transforming growth factor beta-1 (R&D Systems, Minneapolis, MN, USA) to maintain cellular proliferation and multipotency characteristics [10]. Osteogenic differentiation medium contained DMEM with high glucose (4.5 g/l), 10% FBS, 1% antibiotic/antimycotic, 1 nM dexamethasone, 21.6 mg/ml β-glycerophosphate, 50 μg/ml ascorbate-2-phosphate, and 10 μg/ml vitamin D3 (Sigma-Aldrich, St. Louis, MO, USA) [23]. Osteogenic differentiation medium also acted as the priming medium to induce *ALPL* expression prior to sorts. Adipogenic differentiation medium contained DMEM/F-12, 10% FBS, 1% antibiotic/antimycotic, 10 μg/ml insulin, 0.39 μg/ml dexamethasone, 55.6 μg/ml isobutyl-1-methylxanthine (Sigma-Aldrich), and 17.5 μg/ml indomethacin (Cayman Chemical, Ann Arbor, MI, USA) [23]. Chondrogenic differentiation medium contained DMEM with high glucose, 10% FBS, 1% antibiotic/antimycotic, 10 ng/ml transforming growth factor beta-1, 50 μg/ml ascorbate-2-phosphate, 39.0 ng/ml dexamethasone, and 1% insulin –transferrin – selenium + premix (BD Biosciences, San Diego, CA, USA) [24].

#### Adipose-derived stromal cell isolation

All procedures involving human donors were approved by the institutional review board of Rhode Island Hospital. Donors provided consent to use surgical waste material for research purposes. SVF cells were isolated from the subcutaneous adipose tissue of human, female donors (*N* = 4) following established protocols [24]. Briefly, 250 ml liposuction waste tissue was washed with warm phosphate-buffered saline at pH 7.4 and digested with a solution of 0.1% w/v collagenase solution in 1% v/v bovine serum albumin fraction V (Invitrogen, Grand Island, NY, USA) and 2 mM calcium chloride for 60 minutes. Released cells were washed four times with stromal medium, and then incubated for 10 minutes in red blood cell lysis buffer containing 155 mM ammonium chloride, 10 mM potassium carbonate, and 0.1 mM ethylenediamine tetraacetic acid. The resultant cells were then stained with trypan blue and counted using a hemocytometer to determine viability and cell yield. Isolated cells were cryogenically stored in freezing medium containing 10% dimethylsulfoxide, 10% DMEM:Ham’s F12 salt solution, and 80% FBS at a concentration of 5 × 10^6^ to 6 × 10^6^ cells/ml. For designated preliminary/pilot studies, an ASC superlot containing cells from seven nondiabetic donors between the ages of 18 and 60 was purchased commercially and grown to passage 4 before use in experiments (Zen-Bio).

#### Multilineage differentiation

To determine general multipotency of donor cells, primary SVF cells were seeded in 96-well plates (Genesee Biomedical, Denver, CO, USA) at 8,000 to 10,000 cells/well and differentiated down the osteogenic and adipogenic lineages using the differentiation media described previously (*n* = 4 for each lineage and corresponding control). For chondrogenic differentiation, 50,000 cells/well were seeded in a V-bottomed 96-well plate and centrifuged at 400 × *g* to form cell pellets [24]. The cell pellets were then given chondrogenic differentiation medium to induce chondrogenesis or stromal medium to act as a control (*n* = 4). Samples were cultured for either 2 weeks (adipogenic) or 3 weeks (osteogenic, chondrogenic) before being assessed for lineage-specific metabolites as described below.

#### Assessment of osteogenesis, adipogenesis, and chondrogenesis

For osteogenesis, samples at 21 days were fixed with 4% paraformaldehyde and stained with alizarin red S, which binds to calcified matrix and is indicative of bone formation (Sigma-Aldrich). For quantification, the dye was eluted using 10% cetylpyridinium chloride, and the absorbance of the eluent was measured at 540 nm. For adipogenesis, samples at 14 days were fixed and stained with oil red O (Sigma-Aldrich), a dye that binds intracellular lipids indicative of fat formation. For quantification, the stain was eluted from fixed cells using 100% isopropanol, and the absorbance of the eluents was measured at 500 nm. To report elution data on a per-cell basis, cell numbers in each sample were quantified by counting Hoechst 33342-stained nuclei per sample using either Gen5 (BioTek U.S., Winooski, VT, USA) or CellProfiler software [25]. For chondrogenesis, samples at 21 days were digested with 125 μg/ml papain at 65°C and pH 6.5 for 24 hours (Sigma-Aldrich). The sulfated glycosaminoglycan (sGAG) content of each digested pellet was quantified using the dimethylmethylene blue assay, modified from established protocols [26, 27]. Briefly, 2.1 mg dimethylmethylene blue was dissolved in 1 ml of 100% ethanol and 10 ml of 0.3 M HCl containing 304 mg glycine and 237 mg sodium chloride. The resulting solution was brought to a volume of 100 ml with deionized water, and the pH of the dimethylmethylene blue dye solution was adjusted to 1.5 using 6 M HCl to account for nonstandard DNA content contributions across samples [28]. Then 200 μl dye was added to 50 μl digest solution, and the absorbance of the resulting mixture was measured at 525 nm. The PicoGreen assay (Invitrogen, Carlsbad, CA, USA) was used to quantify DNA amounts using 100 μl each digest following the manufacturer’s protocol (480 nm excitation, 520 nm emission). A standard curve was used to calculate the total sGAG amount in each pellet, which was then normalized on a per-DNA basis.

### Beacon design, gene expression-based sorting, and differentiation of sorted cells

#### ALPL molecular beacon design, osteogenic priming, and molecular beacon treatment

**Figure 1:**
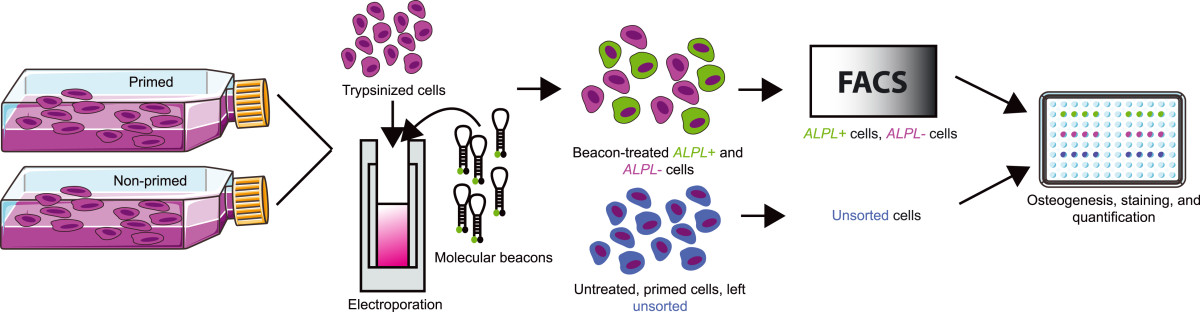
**Alkaline phosphatase liver/bone/kidney-based sorting method.** Gene expression-based sorting of stromal vascular fraction (SVF) cells involved a 4-day osteogenic priming period prior to sorting. Next, cells were treated by electroporation with molecular beacons targeting alkaline phosphatase liver/bone/kidney (*ALPL*) mRNA. The beacon-treated cells were sorted using fluorescence-activated cell sorting (FACS) into *ALPL*+ and *ALPL*– populations. A subset of the initial, primed, SVF cells was left unsorted. The *ALPL*+, *ALPL*–, and unsorted cells were seeded onto tissue culture plates, differentiated down the osteogenic lineage, and assessed for production of calcified matrix.

A custom-designed molecular beacon targeting human *ALPL*, an early marker of osteogenesis [29], was developed as part of our previous work [22]. The beacon sequence was (stems italicized): 5′-(6-carboxyfluorescein) *CGCTCC* AGAGTGTCTTCCGAGGAGGTCAA *GGAGCG* (Black Hole Quencher 1)-3′ (melting temperature, 69.4°C; Eurofins MWG Operon, Huntsville, AL, USA). Freshly thawed, primary SVF cells (and passage 4 superlot ASCs, for pilot studies) were seeded in monolayer at 33,000 cells/cm^2^ and given either osteogenic differentiation medium to prime osteogenic gene expression (primed cells) or expansion medium to maintain their undifferentiated state (nonprimed cells). After 4 days, primed and nonprimed cells were trypsinized using 0.25% trypsin–ethylenediamine tetraacetic acid (Fisher Scientific) and separately resuspended in nonsupplemented base medium (DMEM:Ham’s F12 salt solution) at a concentration of 1 × 10^6^ cells/100 μl. *ALPL* molecular beacons were added to both cell suspensions at a final concentration of 1 μM immediately prior to electroporation. Beacon-treated cells were electroporated using an Amaxa Nucleofector according to manufacturer’s instructions (program U-23; Lonza AG, Basel, Switzerland). When the process was complete, the cuvette was removed and gently rinsed with 500 μl stromal medium three times to collect all cells in a total of 1.5 ml medium. The cells were allowed to recover for 60 minutes in a humidified 37°C, 5% carbon dioxide incubator. Cells were then pelleted by centrifugation at 400 × *g* for 5 minutes and subsequently resuspended at a concentration of 10 × 10^6^ cells/ml in warm Hank’s Buffered Salt Solution (Fisher Scientific) for FACS. Samples were protected from light prior to sorting. All sorts were initiated within 1 hour of electroporation. The overall experimental design for the study is illustrated in Figure 1.

#### Gene expression-based fluorescence-activated cell sorting

SVF cells were sorted based on positive versus negative signals for *ALPL* using the previously mentioned molecular beacon. All gene expression-based sorts were performed on a BD FACSAria IIu instrument (BD Biosciences). Cell samples (primed and nonprimed) treated with *ALPL* beacon were sorted following standard FACS protocols. The instrument was outfitted with an extra-wide 100 μm nozzle to minimize cellular shear stress during the sorting procedure. The forward scatter threshold was set at 5,000 units. Cells were sorted by FACS into positive (*ALPL+*) and negative (*ALPL–*) populations using a 488 nm laser and a 530/30 bandpass filter for detection. In every sorting experiment, cells that were not treated with any molecular beacons were used to set gates defining threshold fluorescence levels (that is, all untreated cells were negative, and all intensities above that level were positive). Primed, unsorted cells from the initial SVF population that were not treated with beacons were used as controls for the study. Nonprimed, unsorted cells were not analyzed as part of the main study; however, that condition was analogous to the general multipotency tests conducted for each donor. A mock sort was also conducted with Donor 4 SVF cells using only forward and side scatter parameters to demonstrate that the initial gating process had no influence on osteogenesis (see Additional file 1). Sort data were analyzed using FlowJo FACS analysis software (TreeStar, Inc., Portland, OR, USA).

#### Cell seeding and differentiation

Following FACS, *ALPL+/-* cells and unsorted cells were seeded in 96-well plates at 8,000 to 10,000 cells/well and differentiated down the osteogenic lineage using the differentiation medium described previously (*N* = 4, *n* = 16 each for osteogenic and control conditions for primed *ALPL*+ cells, primed *ALPL*– cells, nonprimed *ALPL–* cells, and unsorted cells; *N* = 2, *n* = 8 for nonprimed *ALPL*+ cells). After 21 days, samples were fixed, stained, and quantified as described above. Calcified matrix deposition was determined per sample, and then normalized within donor groups to allow for relative comparisons among sorted cell populations. Specifically, raw absorbance values for samples within each donor were normalized to the absorbance of their corresponding unsorted cells. When not noted otherwise, results in this study are reported using these per-sample, donor-normalized values. For a subset of analyses, the raw absorbance values were also normalized on a per-cell basis by counting the number of Hoechst-stained nuclei per sample. In an additional experiment, primed *ALPL*+/- cells and unsorted cells from a representative donor (Donor 1) were differentiated down the osteogenic, adipogenic, and chondrogenic lineages to assess multipotency of the sorted populations (*n* = 4 per subpopulation for osteogenesis, adipogenesis, and corresponding control; *n* = 3 per subpopulation for chondrogenesis and corresponding control).

### Surface marker-based sorting comparison

#### Surface marker-based fluorescence-activated cell sorting

SVF cells from a representative donor (Donor 1) were freshly thawed, stained with trypan blue, and counted with a hemocytometer to determine viability. Cells were then washed twice in 4°C wash buffer (1× PBS, 1% bovine serum albumin), resuspended, and incubated in 4°C blocking buffer (1× PBS, 3% bovine serum albumin) for 10 minutes. Following a wash, cells were aliquoted into separate tubes at a concentration of 10^5^ cells/100 μl for single color controls, negative controls, and sorting. Preconjugated antibodies from BD Pharmingen against human CD34-FITC (#560942), human CD31-PE (#560983), and human CD45-PE-Cy5 (#560974) were used to target the ASC subpopulation [30, 31]. The cells were incubated with antibodies on ice for 20 minutes, followed by a wash. The sort was performed on a BD FACS Influx, and cytometry data were analyzed using FlowJo. Cells were collected into a tube containing expansion medium with 20% FBS.

#### Differentiation of surface marker-sorted cells

Sorted CD34^+^/CD31^-^/CD45^-^ cells were plated at a density of 10,000 cells/well in a tissue culture-treated 96-well plate. The cells were differentiated down the osteogenic and adipogenic lineages (*n* = 4 for each lineage and corresponding controls), and differentiation was quantified as described previously. Results were compared with *ALPL*-based sorting to evaluate the relative effectiveness of the two enrichment techniques.

### Statistical analysis

Significance in multipotency assessments for all four donors was assessed using Student’s *t* test (*P* <0.05; differentiated vs. control in osteogenic, adipogenic, and chondrogenic conditions). Donor-specific experiments were done iteratively to incorporate any systematic, run-to-run error that might be present. Experiments involving *ALPL*-sorted cells differentiated down the osteogenic, adipogenic, and chondrogenic lineages were assessed using two-way analysis of variance (*P* <0.05; *ALPL* expression and differentiation condition) with Holm–Sidak *post hoc* analyses on SigmaPlot software (SYSTAT Software, San Diego, CA, USA). Lineage-specific differentiation response for surface marker-sorted cells was assessed using Student’s *t* test (*P* <0.05; differentiated vs. control in osteogenic and adipogenic conditions). Data are shown as mean ± standard deviation.

## Results

### Stromal vascular fraction isolation, cell yield, viability, and multipotency

SVF cells were isolated from the fat tissue of four human female donors with high yields and viabilities (Table 1). SVF cells from all four donors exhibited multipotency down at least two different lineages. Donors 1 to 3 could successfully differentiate down the osteogenic, adipogenic, and chondrogenic lineages, whereas Donor 4 did not successfully undergo chondrogenesis.

**Table 1:** **Human lipoaspirate donor information**

Donor number	Previous diagnosis	Cell yield (×10 ^6^ cells)	Viability (%)
Donor 1	Breast cancer	87	74
Donor 2	Breast cancer	84	80
Donor 3	Breast cancer	99	95
Donor 4	Skin laxity	111	89
Average		95 ± 12	84 ± 9

Female donors ranging in age from 44 to 62 years old (mean ± standard deviation, 51 ± 8). 250 mL of tissue processed per donor.

### *ALPL*-based sorting of stromal vascular fraction cells

**Figure 2:**
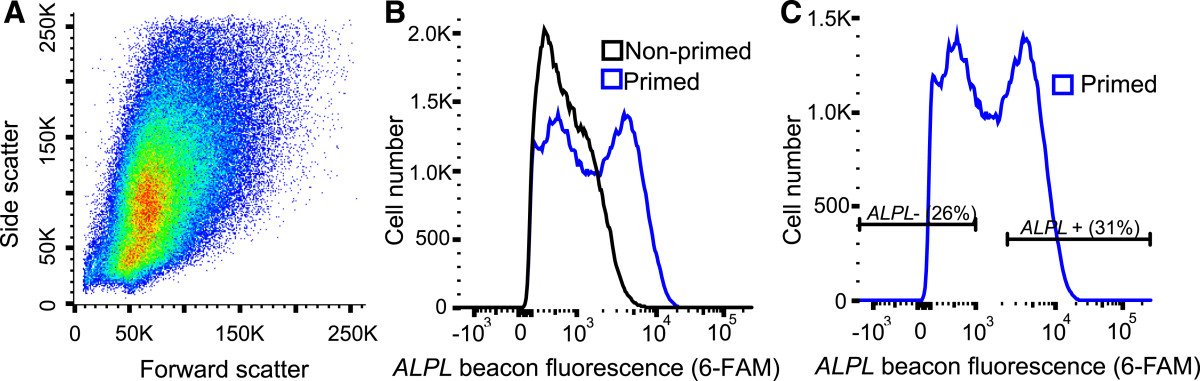
**Alkaline phosphatase liver/bone/kidney sort yields.** Stromal vascular fraction cells treated with alkaline phosphatase liver/bone/kidney (*ALPL*) molecular beacon were sorted based on gene expression signals. **(A)** Cells were first gated using forward and side scatter measurements to eliminate debris and cellular aggregates. **(B)** Nonprimed cells were largely *ALPL*–, displaying a unimodal distribution overlapping with the primed *ALPL*– peak. **(C)** Sorting was determined using the bimodal distribution of primed SVF cells, with clear peaks existing for positive expressers (*ALPL+*) and negative expressers (*ALPL–*). A representative set of data is shown here for a single sorting experiment. 6-FAM, 6-carboxyfluorescein.

SVF cells were successfully sorted based on *ALPL* gene expression using a custom designed molecular beacon, resulting in an average cell yield for primed *ALPL*+ cells of 9 ± 3% of the input population (see Additional file 2). Following standard FACS procedures, the input cell population was gated using forward and side scatter parameters, which eliminated aberrantly large or small events (debris particles and cell aggregates; Figure 2). For this gated population, sort data showed that, on average, 34 ± 12% of cells displayed a positive beacon signal (*ALPL+*) while 18 ± 7% had low/no signal (*ALPL–*; see Additional file 2). To obtain distinct sort populations, a gap was inserted between positive and negative groups (Figure 2), which represented 48 ± 9% of the gated cells (see Additional file 2). Decreasing the size of this gap would improve cell yields but might result in lower purity for *ALPL+/-* sorted populations.

### Osteogenic differentiation across *ALPL*-sorted donors

**Figure 3:**
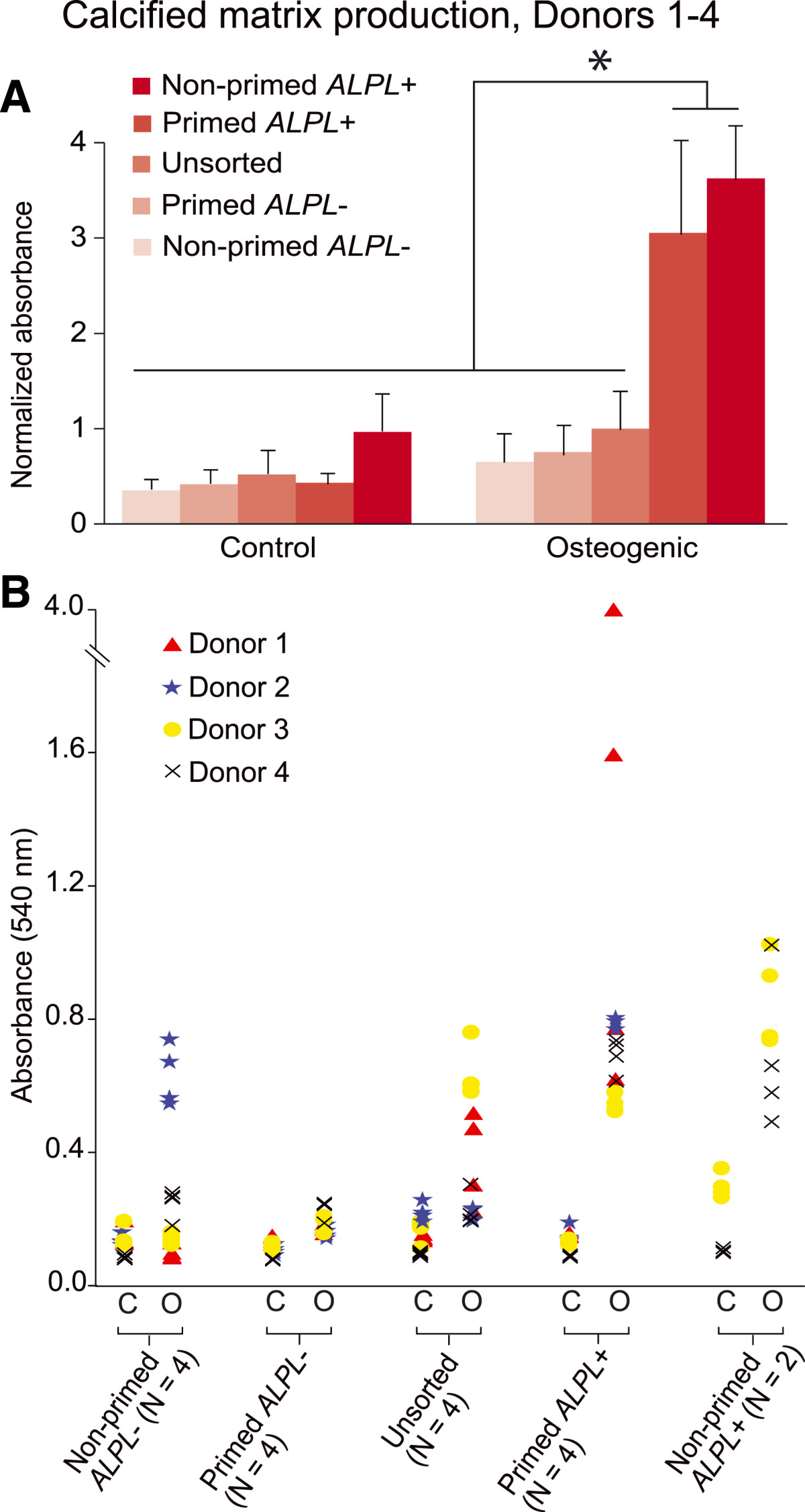
**Osteogenic differentiation of alkaline phosphatase liver/bone/kidney-sorted stromal vascular fraction cells. (A)** Osteogenic *ALPL*+ cells produced significantly more matrix than any other group. Raw absorbance values for all groups were normalized within donors to their respective osteogenic, unsorted samples to allow for relative comparisons. **(B)** While trends among sorted groups remained the same when separating data by donor, extensive variability in matrix production was observed, with some donor cell populations being especially productive and others relatively dormant. Two-way analysis of variance with Holm–Sidak *post hoc* test determined significance among sorted groups (*P <0.05). *ALPL*, alkaline phosphatase liver/bone/kidney.

**Figure 4:**
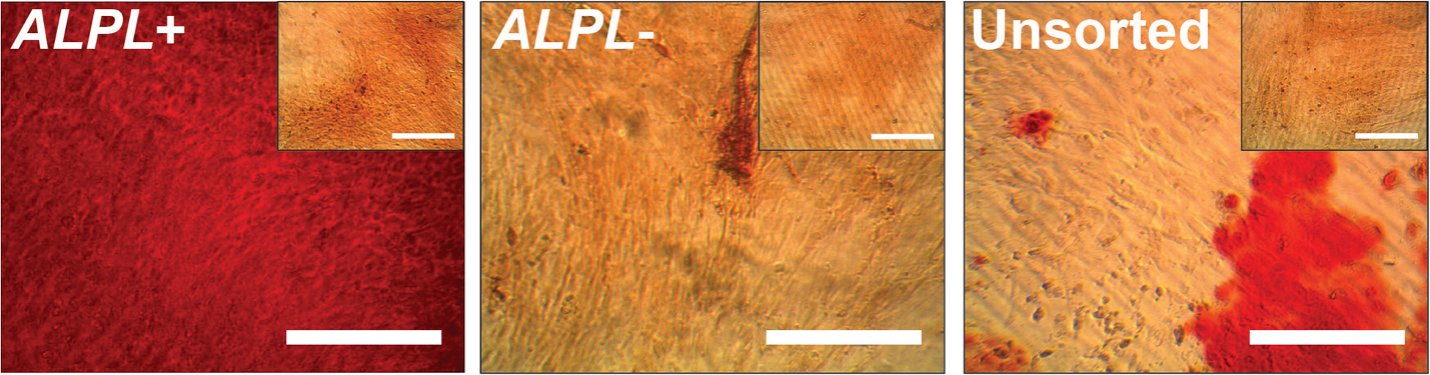
**Alizarin red S staining of osteogenically primed, sorted stromal vascular fraction cells.** Primed, sorted SVF cells were differentiated down the osteogenic lineage for 3 weeks and stained for calcified matrix deposition (control medium conditions shown in insets). *ALPL*+ cells visually produced the most matrix, with very dense, consistent staining across the entire sample. *ALPL*– cells produced the least matrix, with the stain appearing as a slight wash of orange–red across the culture surface. Unsorted cells produced some matrix, but deposition was scattered, with some areas appearing very red and others unstained. Scale bar: 100 μm. *ALPL*, alkaline phosphatase liver/bone/kidney.

*ALPL+* cells deposited dramatically more calcified matrix than unsorted or *ALPL–* cells under osteogenic conditions (Figure 3A). In particular, primed *ALPL+* cells induced for osteogenesis displayed a strong propensity for osteogenic matrix formation, showing 2.1-fold more calcified matrix deposition than unsorted cells (*P* <0.05) and 3.7-fold more than primed *ALPL–* cells (*P* <0.05). While significant variation existed among the four donors, these trends were generally consistent, with *ALPL+* samples exhibiting more robust osteogenic responses (Figure 3B; see Additional file 3). Unsorted samples did successfully differentiate and produce calcified matrix, but the extent of matrix formation on a donor-normalized basis was much less than that of the primed *ALPL+* samples (Figure 4). Unsorted cells deposited 0.9-fold more matrix than their matched, undifferentiated controls, whereas primed *ALPL*+ cells deposited 5.1-fold more matrix than their undifferentiated controls. Normalizing matrix deposition on a per-cell basis revealed the same trends, suggesting that the sorting process was successfully isolating individual cells with increased synthetic capacity (Table 2).

**Table 2:** **Raw osteogenic data for sorted and unsorted cell populations**

	Cell numbers/sample (×10 ^3^)		Absorbance/sample		Absorbance/10,000 cells	
	Control	Osteogenic	Control	Osteogenic	Control	Osteogenic
Nonprimed *ALPL+*	13.5 ± 4.2	11.2 ± 9.0	0.21 ± 0.10	0.80 ± 0.21	0.15 ± 0.04	2.71 ± 4.76
Primed *ALPL+*	9.5 ± 3.2	11.0 ± 4.0	0.14 ± 0.03	1.03 ± 0.87	0.16 ± 0.04	0.98 ± 0.64
Unsorted	11.5 ± 3.4	15.0 ± 8.7	0.16 ± 0.05	0.36 ± 0.19	0.14 ± 0.03	0.60 ± 0.81
Primed *ALPL–*	7.4 ± 1.6	14.0 ± 4.7	0.11 ± 0.02	0.19 ± 0.04	0.15 ± 0.02	0.14 ± 0.03
Nonprimed *ALPL–*	9.2 ± 2.5	13.0 ± 5.5	0.13 ± 0.03	0.28 ± 0.22	0.15 ± 0.03	0.19 ± 0.11

Primed indicates cells received osteogenic differentiation medium prior to alkaline phosphatase liver/bone/kidney (*ALPL*) sorting. Unsorted population was primed. Data for control and osteogenic media conditions are shown (mean ± standard deviation).

### *ALPL*-based sorting and differentiation of passaged human adipose-derived stem/stromal cells

Monolayer-expanded, passage 4 superlot ASCs were sorted based on *ALPL* expression and differentiated down the osteogenic lineage as described previously. The yield of *ALPL+* cells with respect to overall cell numbers was 6.7%, with the forward and side scatter gated population being comprised of 81% *ALPL*+ cells and 12% *ALPL*– cells. Following osteogenic differentiation, primed *ALPL*+ cells displayed a 1.2-fold increase in matrix deposition over unsorted cells (*P* <0.05); however, raw absorbance values reflecting total calcified matrix amounts per sample were on average one-tenth that observed for freshly isolated SVF cells across all sorted groups and donors.

### Multipotency assessment of *ALPL*-sorted cells

**Figure 5:**
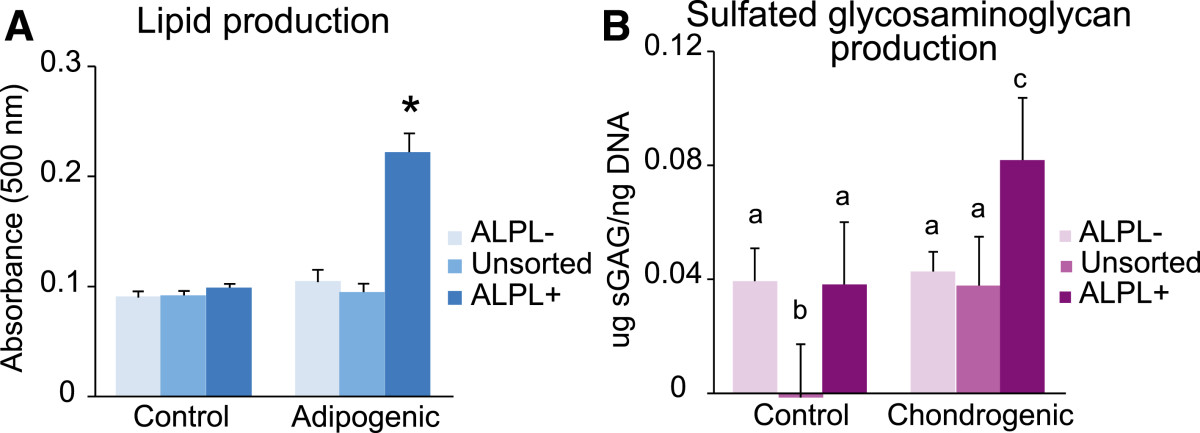
**Multilineage differentiation of alkaline phosphatase liver/bone/kidney-sorted stromal vascular fraction cells.** Primed, sorted stromal vascular fraction subpopulations were differentiated down the adipogenic and chondrogenic lineages to determine multipotency. *ALPL*+ samples showed significant increases in metabolite production over *ALPL*– and unsorted samples for both **(A)** adipogenic and **(B)** chondrogenic conditions. Of particular note, only *ALPL*+ samples showed a significant differentiation response for adipogenesis. *ALPL–* samples showed no response for either lineage, and unsorted samples only differentiated for chondrogenesis. Two-way analysis of variance with Holm–Sidak *post hoc* tests determined significance (*or nonmatching letters, *P* <0.05). *ALPL*, alkaline phosphatase liver/bone/kidney; sGAG, sulfated glycosaminoglycan.

To determine whether *ALPL* sorting isolated a unipotent or multipotent cellular phenotype, additional lineages beyond osteogenesis were assessed for primed *ALPL+/-* and unsorted groups. *ALPL+* cells were the only population capable of adipogenesis (*P* <0.05; Figure 5A), with lipid accumulation in unsorted and *ALPL–* samples not changing significantly (*P* = 0.63 and *P* = 0.053, respectively). *ALPL+* cells showed a 1.3-fold increase in lipid formation over unsorted cells (*P* <0.05). Chondrogenesis was assessed by quantifying sGAG content normalized to DNA. Both *ALPL+* and unsorted samples showed successful differentiation responses (*P* <0.05), whereas *ALPL–* samples did not (*P* = 0.79; Figure 5B). *ALPL+* cells produced 1.2-fold and 0.9-fold more sGAG than unsorted cells and *ALPL–* cells, respectively (*P* <0.05).

### Surface marker-based sorting and differentiation of stromal vascular fraction cells

SVF cells were sorted using a traditional, surface marker-based approach targeting the CD34^+^/31^-^/45^-^ ASC subpopulation and then differentiated down the osteogenic and adipogenic lineages. CD34^+^/31^-^/45^-^ cells represented 4% of the overall SVF population and 14% of the gated cell population (see Additional file 4). Following differentiation, the CD34^+^/31^-^/45^-^ osteogenic samples produced significantly less matrix than control samples on a per-sample basis, indicating an unsuccessful differentiation response (absorbance: 0.19 ± 0.02 vs. 0.23 ± 0.02, *P* <0.02; see Additional file 4). On a per-cell basis, osteogenic samples had more matrix deposition than controls, but this increase did not achieve significance (absorbance: 0.22 ± 0.09 vs. 0.14 ± 0.02, *P =* 0.16). CD34^+^/31^-^/45^-^ adipogenic samples produced significantly more lipids than control samples, indicating successful adipogenesis (absorbance: 1.82 ± 0.31 vs. 0.58 ± 0.13, *P* <0.05), and when normalized on a per-cell basis the relationship remained the same, with adipogenic cells producing significantly more matrix than control cells (absorbance/cell: 4.47 ± 0.90 vs. 0.39 ± 0.11, *P* <0.05).

## Discussion

The results of this study showed that *ALPL*+ cell populations undergoing osteogenesis could produce up to 4.9 times the calcified matrix of unsorted samples, while also exhibiting increased differentiation potential for adipogenic and chondrogenic lineages. We predicted that, given the heterogeneous nature of SVF cells, only a small subset would express *ALPL* in response to osteogenic priming. However, results showed that, on average, 34% of gated cells in the SVF were capable of expressing the early osteogenic marker, indicated by a positive *ALPL* molecular beacon fluorescence. Calcified matrix deposition was significantly increased in *ALPL+* cells compared with both unsorted and surface marker-sorted stromal cells, and these trends remained the same whether they were assessed on a per-sample or per-cell basis. *ALPL*+ cells showed increases in adipogenic and chondrogenic potential as well, indicating that they are not only multipotent but represent a beneficial subpopulation for lineages beyond osteogenesis. Further, this *ALPL*+ subpopulation was isolated with significantly higher yields than traditional stem cell sorting approaches, representing a potentially transformative method of cell enrichment for MSCs.

Fluorescent tagging of live-cell gene expression in this study allowed for enrichment of SVF cells in a manner similar to surface marker-based sorting. Gene expression-based sorting is an advantageous method for cell enrichment for several reasons. Because gene expression occurs before protein translation, we are able to target cells earlier in the differentiation process than is feasible with protein labeling. This paradigm-shifting approach had no negative effect on cellular growth and differentiation, and the *ALPL*-targeting probe is safely degraded by natural processes in the cells [22]. Most importantly, *ALPL*+ cells were found to be a highly productive subpopulation within the SVF and could be an excellent cell source to target for regenerative therapies and basic research. To gain further insight into the abilities and cellular composition of *ALPL+* subpopulations, we tested the multilineage differentiation capability of primed, sorted samples. Presumably, sorting based on osteogenic gene expression after a 4-day priming period would result in a population of cells geared towards osteogenic differentiation. As such, we hypothesized that *ALPL+* subpopulations would have diminished multilineage differentiation capabilities. Interestingly, *ALPL+* subpopulations showed significant increases not only in osteogenic metabolite production but also in characteristic chondrogenic and adipogenic molecules production. When subjected to a standard, 2-week adipogenic differentiation protocol, *ALPL+* samples produced 2.3 times the amount of lipid compared with unsorted samples. For chondrogenesis, *ALPL+* samples possessed one-half the DNA content of unsorted samples while producing the same amount of sGAG, suggesting that *ALPL+* cells were potentially producing double the sGAG per cell than unsorted cells. These findings demonstrated that *ALPL+* subpopulations have increased multilineage differentiation capabilities, although the most notable improvement was still for osteogenesis. Further, we found that a 4-day priming period did not fully commit cells to the osteogenic lineage, which is consistent with previous reports [32].

Although *ALPL*-based sorting reliably isolates cells capable of increased calcified matrix deposition, it is unclear whether the procedure captures cells with the greatest synthetic potential or simply removes an inhibitory population. If we isolated maximally synthetic cells, then an additive relationship should be apparent among sorted groups, with the output of the unsorted population approximately equaling the sum of its contributing subpopulations, primed *ALPL+/-* cells. Surprisingly, increases in matrix production in *ALPL+* cells did not linearly relate to the percentage of cells removed from the initial population. In *ALPL+* samples, removing nonexpressing cells, which represented about 18% of the SVF cells on average, resulted in a 210% increase in matrix production. This disproportionate increase may be due to beneficial intercellular communication among cells capable of differentiation, which are enriched in *ALPL+* cell populations compared with *ALPL–* and unsorted samples. This hypothesis is consistent with studies showing that osteogenic differentiation in MSCs is dependent on intercellular communication [33–35]. In comparison, matrix deposition by *ALPL–* cells was less than that by unsorted cells, but this difference was not statistically significant, indicating the cells still had some osteogenic capacity and were probably not actively inhibiting the process. The specific role of intercellular communication within the sorted populations is yet to be examined.

Donor-to-donor variability is a continual obstacle in assessing the robustness of a treatment or experimental finding [36, 37], so in this study we sorted cells from four different donors to demonstrate the broad applicability of the enrichment procedure. As expected, each donor population deposited variable amounts of calcified matrix following osteogenic differentiation, with Donor 4 cells being the highest producers overall. Interestingly, unsorted Donor 3 samples were unable to osteogenically differentiate on a per-sample basis, but the *ALPL+* subpopulation overcame this lack of response with a robust, successful differentiation. Similarly, while unsorted Donor 1 samples were unable to differentiate significantly down the adipogenic lineage, contrary to results from initial multipotency testing on this donor, the *ALPL+* subpopulation exhibited a positive differentiation response, indicating superior differentiation capabilities of these sorted cells. Despite differences among donors, gene expression-based sorting consistently isolated highly productive subpopulations of cells (*ALPL+*). The versatility of this procedure is apparent regardless of innate donor differentiation ability.

Two of the donor SVF populations (Donors 3 and 4) contained a subpopulation of cells that inherently expressed *ALPL*, without any presort priming. Interestingly, nonprimed *ALPL +* cells performed similarly to primed *ALPL+* cells in terms of matrix deposition, suggesting that some donors may have a ready population of high-potential osteogenic cells that can be isolated directly without need for priming. Another contributor to variability may be the morbidities associated with each donor. Notably, Donors 1, 2, and 3 had previous breast cancer diagnoses, while Donor 4 did not. Donor 4 cells displayed more robust osteogenic differentiation than the others, perhaps due to being from a noncancerous donor. Regardless of prior morbidity, all of the donors were found capable of multilineage differentiation. This is especially encouraging, because it suggests that even prior pathology and treatment regimens did not limit the differentiation ability of *ALPL+* subpopulations. That said, more detailed medical histories would be necessary to make any specific hypotheses as to a donor-specific cause of any differentiation differences. Future studies focusing on donor population characteristics may help answer some of these questions.

Surface marker-based sorting strategies are the gold standard technique for cell enrichment. However, these approaches typically produce cell yields significantly lower than those obtained via our gene expression-based sorting strategy. While the highest, reported MSC yields for antigen-based sorting are ~30% of the gated population, some of the more specific surface marker profiles limit yields to <0.1% [17, 31, 38–41]. Also, in many cases the assessed population may only represent a small portion of the initial cell harvest because of restrictive gating for size and granularity. In the current study, we also included a gap region between positive and negative *ALPL* population peaks to minimize contamination from the other group. Because the peaks are often relatively near each other, a large proportion of cells are sacrificed to the gap (24 to 62% of cells). Refinements to the procedure, such as using a more intense fluorophore with better quenching, can further separate positive and negative peaks, lessening the number of cells present in the gap region. Even with these limitations, the *ALPL-*based sorting approach produced an overall 9% yield for *ALPL+* cells (34% of gated events). We also performed a representative surface marker-based sorting experiment, which isolated cells using a broad CD34^+^/CD31^-^/CD45^-^ immunophenotype profile for ASCs [30]. Using this loose definition, ASCs represented only 4% of the population, which is two-fifths of the yield we obtained via gene expression-based sorting. In addition to limited yield, CD34^+^/CD31^-^/CD45^-^ cells displayed no increase in calcified matrix deposition when compared with unsorted cells. We hypothesized that surface marker-based sorting would isolate tightly defined, highly differentiable subsets of the larger SVF population. Surprisingly, we found that these cells demonstrated a more limited ability to differentiate down the osteogenic lineage than *ALPL+* or unsorted cells. Because of the rigorous, immunophenotype definitions characteristic of surface marker-based sorting, it is possible that other subpopulations of cells capable of differentiation are excluded, resulting in diminished osteogenic capabilities [13]. The broad inclusiveness of our gene expression-based sorting procedure ensures that all cells capable of osteogenic gene expression are captured, rather than a single immunophenotype. Others have shown that a mixed population of cells may be more beneficial to osteogenesis than a highly purified stem-like population [42–44]. These results bolster the notion that stem cell-specific sorting, which aims to eliminate nonstem immunophenotypes, may actually impair the overall regenerative potential of heterogeneous MSC populations.

In addition to experiments using primary cells, gene expression-based sorting was also performed with highly enriched, passaged ASCs comprised of a superlot of donors. Researchers have shown that passaging freshly isolated SVF cells results in enrichment for highly adherent stem-like cells, since less adherent cell types, such as endothelial and circulating hematopoietic cells, remain detached from the culture surface [9, 13]. Based on the reported homogeneity of these passaged ASC populations, we hypothesized that the majority of these cells would express *ALPL* in response to osteogenic growth factors and that increases in osteogenic matrix deposition in *ALPL+* cells compared with unsorted cells would be minimal. As predicted, a larger proportion of cells exhibited a positive beacon signal (81%) based on flow cytometry. Interestingly, a significant increase in matrix deposition was still observed for *ALPL+* cells versus unsorted samples. This observation was surprising because it indicated that even supposedly pure ASC populations could be further enriched for cells with enhanced osteogenic capabilities. Despite the positive trends, however, superlot ASCs differentiated poorly in comparison with freshly isolated cells, producing 10 times less matrix than either sorted or unsorted primary SVF cells. Other researchers have also acknowledged the drawbacks of using passaged stromal cells for differentiation [45–47]. While the gene expression-based sorting procedure does benefit the overall differentiation response of passaged ASCs, our best results were achieved using primary SVF-derived cells.

The gene expression-based sorting strategy demonstrated in this study requires a 4-day osteogenic priming period, which is nonideal for single-surgery therapeutic applications. However, priming has been shown to be an effective approach for inducing the expression of characteristic molecules [48]. That said, refinements to our strategy, such as targeting mRNA transcripts earlier in the gene expression pathway, should be considered to minimize or eliminate the priming period. However, the increase in matrix production currently afforded to sorted cells may offset drawbacks associated with cell priming, and some donors may not even require this if *ALPL+* cells are inherently present at sufficient numbers. Another potential drawback of the sorting procedure is the presence of false positive cells in the *ALPL+* population, given that nonspecific fluorescence is a known limitation of molecular beacons [22, 49, 50]. Despite this potential source of error, *ALPL +* populations still produced significantly more matrix than any other population of SVF-derived cells, suggesting minimal negative influence from the inclusion of some false-positive cells. Improvements in beacon technology have the potential to further improve outcomes by minimizing this source of error. Lastly, the sorting procedure functions best when targeting abundant mRNA molecules, because the high concentration of target transcripts yields an easily defined positive signal. To target genes that are expressed in low copy numbers, a different strategy must be employed, with one possibility being binding multiple probes to a single transcript to amplify positive signals within the cell [51].

## Conclusion

We have demonstrated that sorting stromal cells based on expression of *ALPL* mRNA isolated a highly synthetic *ALPL +* subpopulation with significantly improved osteogenesis capabilities. These cells produced more calcified matrix than unsorted stromal cells and were collected in higher yields than existing cell enrichment strategies. *ALPL+* cells also showed improved multilineage differentiation capability over unsorted samples, indicating that they are most probably a highly responsive subpopulation within the SVF and not merely osteoprogenitors. Furthermore, since this method improves the cell source at the front end of tissue engineering and cell-based therapies, downstream outcomes have the possibility to see even greater gains. The significance of this work lies in a potential paradigm shift in how cells can be identified and sorted using gene expression-based markers with existing flow cytometry infrastructure. Both basic science investigations and cell-based, clinical therapies could realize significant improvements for applications involving heterogeneous samples.

### Note

This article is part of an ‘Emerging Investigators’ collection showcasing the work of early career investigators who have demonstrated growing leadership in the field of stem cells and regenerative medicine. Other articles in the series can be found online at http://stemcellres.com/series/emerginginvestigators.

## Electronic supplementary material

Additional file 1: Figure S1: Showing a mock sort of gated SVF cells. Donor 4 SVF cells were gated and sorted based on just forward (FSC) and side (SSC) scatter parameters, to ensure that the initial gating process alone was not enriching the cells. Gated osteogenic samples did not show any change in calcified matrix production compared with unsorted osteogenic samples, providing evidence that simply putting cells through the flow cytometer did not affect osteogenic response to any significant extent (*P* = 0.89). (PDF )

Additional file 2: Table S1: Presenting donor-specific yields for the *ALPL*-sorting procedure. (DOC )

Additional file 3: Figure S2: Showing donor-specific calcified matrix production for sorted SVF cells. SVF cells from four, distinct donors were sorted based on expression of *ALPL* mRNA. Absorbance values of eluted alizarin red S, indicative of calcified matrix production, were normalized on a per-cell basis by counting Hoechst 33342-stained nuclei in each sample. In all donors, *ALPL+* groups consistently produced more calcified matrix on a per-cell basis than any other group. However, in Donor 4 the overall matrix production was higher, and so primed *ALPL+* cells were not significantly more productive than unsorted cells (*P* = 0.97). Sample groups with nonmatching letters are significantly different (*P* <0.05). (PDF )

Additional file 4: Figure S3: Showing surface marker-based sorting of SVF cells. Freshly thawed SVF cells were labeled with fluorescent antibodies for CD34, CD31, and CD45 and sorted using a BD FACS Influx. **(A)** Gated forward and side scatter cells were **(B)** 23% positive for CD34 surface antigen expression. **(C)** Of the CD34^+^ cells, 58% were also CD31^-^ and CD45^-^. Overall yield for CD34^+^/31^-^/45^-^ cells was 4%, and these cells displayed only a limited ability to differentiate down the osteogenic lineage. **(D)** In comparison, primed, *ALPL*+ cells produced 25-fold more calcified matrix than CD34^+^/31^-^/45^-^ cells. Sample groups with nonmatching letters are significantly different (*P* <0.05). (PDF )

## Abbreviations

*ALPL*: alkaline phosphatase liver/bone/kidney
ASC: human adipose-derived stem/stromal cell
DMEM: Dulbecco’s modified Eagle’s medium
FACS: fluorescence-activated cell sorting
FBS: fetal bovine serum
MSC: mesenchymal stem cell
sGAG: sulfated glycosaminoglycan
SVF: stromal vascular fraction.
