# Supplementary figures and images for: Gene expression-based enrichment of live cells from adipose tissue produces subpopulations with improved osteogenic potential
Source: Stem Cell Res Ther. 2014 Oct 6;5:145. doi: 10.1186/scrt502 (PMC4619280; doi:10.1186/scrt502)

# Calcified matrix production: FSC/SSC gate-sorted cells

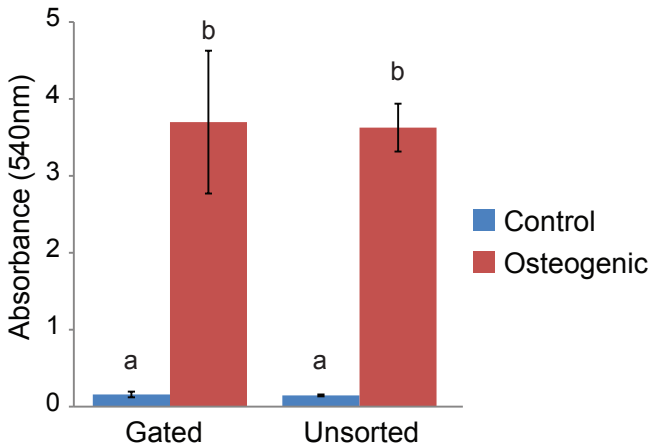

Supplement: Supplementary file 1 — Additional file 1: Figure S1: Showing a mock sort of gated SVF cells. Donor 4 SVF cells were gated and sorted based on just forward (FSC) and side (SSC) scatter parameters, to ensure that the initial gating process alone was not enriching the cells. Gated osteogenic samples did not show any change in calcified matrix production compared with unsorted osteogenic samples, providing evidence that simply putting cells through the flow cytometer did not affect osteogenic response to any significant extent (P = 0.89). (PDF ) [file 13287_2014_410_MOESM1_ESM.pdf]

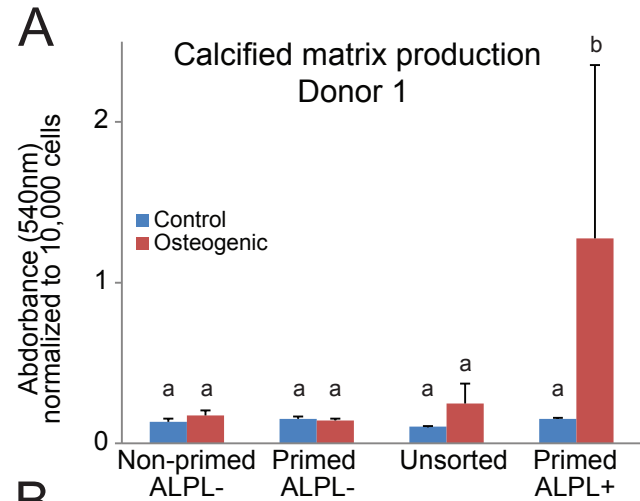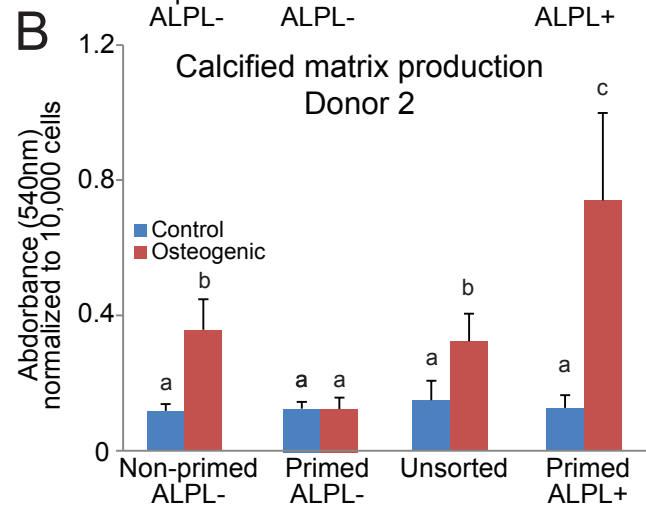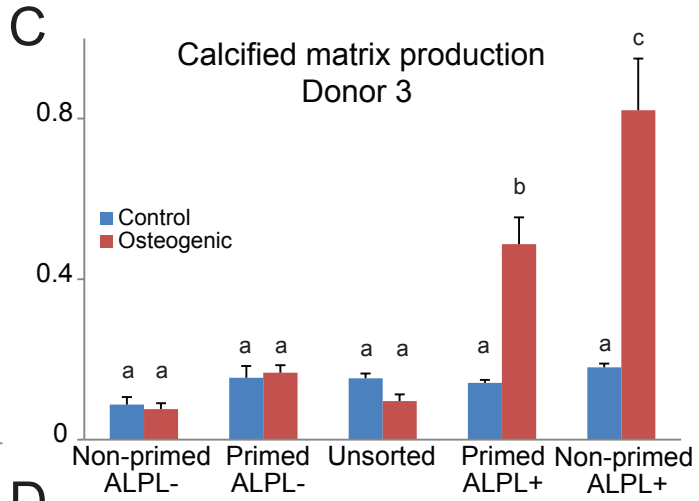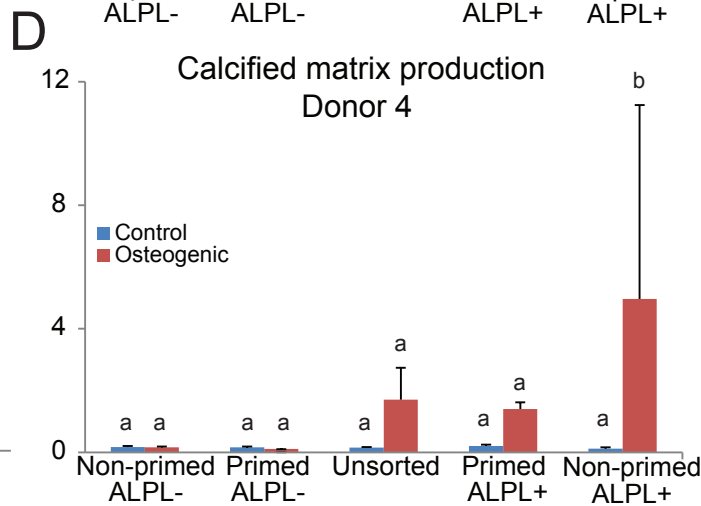

Supplement: Supplementary file 3 — Additional file 3: Figure S2: Showing donor-specific calcified matrix production for sorted SVF cells. SVF cells from four, distinct donors were sorted based on expression of ALPL mRNA. Absorbance values of eluted alizarin red S, indicative of calcified matrix production, were normalized on a per-cell basis by counting Hoechst 33342-stained nuclei in each sample. In all donors, ALPL+ groups consistently produced more calcified matrix on a per-cell basis than any other group. However, in Donor 4 the overall matrix production was higher, and so primed ALPL+ cells were not significantly more productive than unsorted cells (P = 0.97). Sample groups with nonmatching letters are significantly different (P <0.05). (PDF ) [file 13287_2014_410_MOESM3_ESM.pdf]

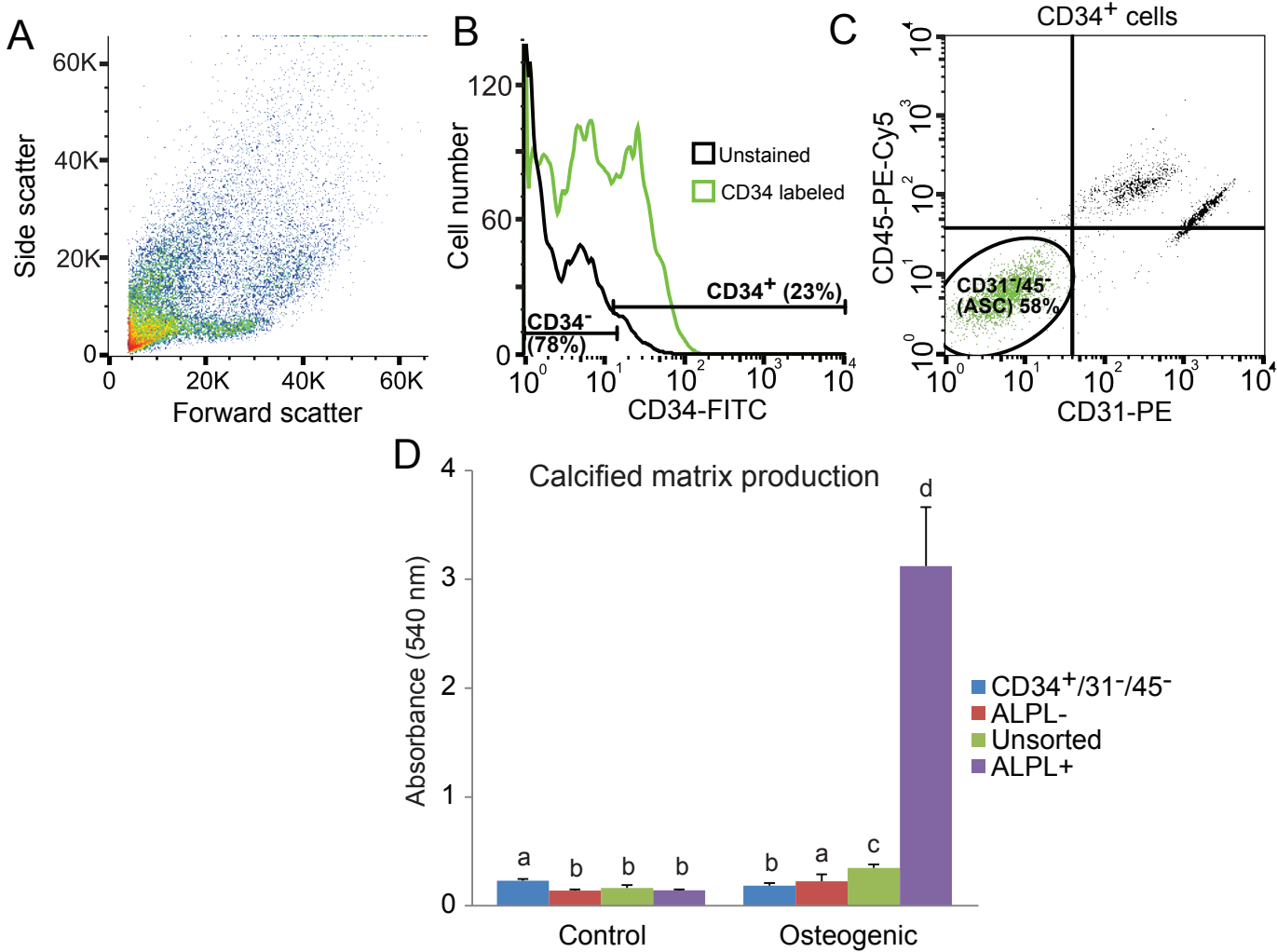

Supplement: Supplementary file 4 — Additional file 4: Figure S3: Showing surface marker-based sorting of SVF cells. Freshly thawed SVF cells were labeled with fluorescent antibodies for CD34, CD31, and CD45 and sorted using a BD FACS Influx. (A) Gated forward and side scatter cells were (B) 23% positive for CD34 surface antigen expression. (C) Of the CD34+ cells, 58% were also CD31- and CD45-. Overall yield for CD34+/31-/45- cells was 4%, and these cells displayed only a limited ability to differentiate down the osteogenic lineage. (D) In comparison, primed, ALPL+ cells produced 25-fold more calcified matrix than CD34+/31-/45- cells. Sample groups with nonmatching letters are significantly different (P <0.05). (PDF ) [file 13287_2014_410_MOESM4_ESM.pdf]
