# Supplementary material for: Gene expression-based enrichment of live cells from adipose tissue produces subpopulations with improved osteogenic potential
Source: Stem Cell Res Ther. 2014 Oct 6;5:145. doi: 10.1186/scrt502 (PMC4619280; doi:10.1186/scrt502)
Supplement: Supplementary file 2 — Additional file 2: Table S1: Presenting donor-specific yields for the ALPL-sorting procedure. (DOC ) [file 13287_2014_410_MOESM2_ESM.doc]

| **Supporting table S1 Donor-specific cell yields for *ALPL*-sorting procedure** | | | | | | | | | | | | | | | | | | | | | | | | | | | | | | | | | | | |
| --- | --- | --- | --- | --- | --- | --- | --- | --- | --- | --- | --- | --- | --- | --- | --- | --- | --- | --- | --- | --- | --- | --- | --- | --- | --- | --- | --- | --- | --- | --- | --- | --- | --- | --- | --- |
|  |  | **Total input cells**  **(x10^5^)** | |  |  | **Cells post-gating**  **(X 10^5^)** | | |  |  | | **Cells post-*ALPL* sorting**  **(X 10^5^)** | | | | |  | | **% yield of input cells** | | | | |  | | **% yield of gated cells** | | | | |  | | **% lost to gap** | | |
|  |  |  |  |  |  |  |  | | |  |  | | **Primed** | | **Non-primed** | | |  | | **Primed** | | **Non-primed** | | |  | | **Primed** | | **Non-primed** | | |  | |  |  |
|  |  | **Primed** | **Non-primed** |  |  | **Primed** | **Non-primed** | | |  |  | | ***ALPL+*** | ***ALPL-*** | ***ALPL+*** | ***ALPL-*** | |  | | ***ALPL+*** | ***ALPL-*** | ***ALPL+*** | ***ALPL-*** | |  | | ***ALPL+*** | ***ALPL-*** | ***ALPL+*** | ***ALPL-*** | |  | | **Primed** | **Non-primed** |
| **DONOR 1** |  | 31 | 36 |  |  | 7.1 | 3.3 | | |  |  | | 2.2 | 1.9 | 0.05 | 2.2 | |  | | 7.1 | 6.2 | 0.1 | 6.1 | |  | | 31 | 27 | 1.5 | 67 | |  | | 42 | 32 |
| **DONOR 2** |  | 58 | 57 |  |  | 6.5 | 2.7 | | |  |  | | 3.2 | 0.6 | 0.06 | 2.0 | |  | | 5.5 | 1.0 | 0.1 | 3.5 | |  | | 49 | 9.2 | 2.2 | 74 | |  | | 42 | 24 |
| **DONOR 3** |  | 24 | 38 |  |  | 7.3 | 12.8 | | |  |  | | 2.8 | 1.0 | 0.4 | 6.1 | |  | | 11.9 | 4.3 | 1.0 | 15.9 | |  | | 38 | 14 | 3.1 | 48 | |  | | 48 | 49 |
| **DONOR 4** |  | 9.3 | 15 |  |  | 4.7 | 8.2 | | |  |  | | 0.9 | 0.9 | 1.2 | 1.2 | |  | | 9.7 | 9.7 | 7.9 | 13.9 | |  | | 19 | 19 | 14.6 | 26 | |  | | 62 | 60 |
| **AVERAGE** |  | 30 ± 20 | 37 ± 17 |  |  | 6.4 ± 1.2 | | 6.8 ± 4.7 | |  |  | | 2.3 ± 1.0 | 1.1 ± 0.6 | 0.4 ± 0.5 | 3.1 ± 2.0 | |  | | 8.6 ± 2.8 | 5.3 ± 3.6 | 2.3 ± 3.8 | 9.9 ± 6.0 | |  | | 34 ± 13 | 18 ± 8 | 5.4 ± 6.2 | 54 ± 22 | |  | | 48 ± 9 | 41 ± 16 |
| Total input of SVF cells to the sorter is shown, followed by the number of cells recovered following forward/side scatter gating. Final primed, *ALPL*+/- subpopulation yields are reported based on input and gated cell numbers. Percentage of cells lost to “gap” region between ALPL+/- peaks is also included. Mean ± standard deviation is reported for the combined donor data. | | | | | | | | | | | | | | | | | | | | | | | | | | | | | | | | | | | |
